# Characterization of the plasma proteome from healthy adult dogs

**DOI:** 10.3389/fvets.2024.1356318

**Published:** 2024-04-04

**Authors:** Pavlos G. Doulidis, Benno Kuropka, Carolina Frizzo Ramos, Alexandro Rodríguez-Rojas, Iwan A. Burgener

**Affiliations:** ^1^Division for Small Animal Internal Medicine, Department for Small Animals and Horses, University of Veterinary Medicine Vienna, Vienna, Austria; ^2^Institute of Chemistry and Biochemistry, Freie Universität Berlin, Berlin, Germany; ^3^The Interuniversity Messerli Research Institute, Medical University Vienna, Vienna, Austria; ^4^Clinical Center for Small Animals, University of Veterinary Medicine Vienna, Vienna, Austria

**Keywords:** plasma proteomics, mass-spectrometry, canine, veterinary medicine, biomarker

## Abstract

**Introduction:**

Bloodwork is a widely used diagnostic tool in veterinary medicine, as diagnosis and therapeutic interventions often rely on blood biomarkers. However, biomarkers available in veterinary medicine often lack sensitivity or specificity. Mass spectrometry-based proteomics technology has been extensively used in the analysis of biological fluids. It offers excellent potential for a more comprehensive characterization of the plasma proteome in veterinary medicine.

**Methods:**

In this study, we aimed to identify and quantify plasma proteins in a cohort of healthy dogs and compare two techniques for depleting high-abundance plasma proteins to enable the detection of lower-abundance proteins via label-free quantification liquid chromatography-mass spectrometry. We utilized surplus lithium-heparin plasma from 30 healthy dogs, subdivided into five groups of pooled plasma from 6 randomly selected individuals each. Firstly, we used a commercial kit to deplete high-abundance plasma proteins. Secondly, we employed an in-house method to remove albumin using Blue-Sepharose.

**Results and discussion:**

Among all the samples, some of the most abundant proteins identified were apolipoprotein A and B, albumin, alpha-2-macroglobulin, fibrinogen beta chain, fibronectin, complement C3, serotransferrin, and coagulation factor V. However, neither of the depletion techniques achieved significant depletion of highly abundant proteins. Despite this limitation, we could detect and quantify many clinically relevant proteins. Determining the healthy canine proteome is a crucial first step in establishing a reference proteome for canine plasma. After enrichment, this reference proteome can later be utilized to identify protein markers associated with different diseases, thereby contributing to the diagnosis and prognosis of various pathologies.

## Introduction

Biomarkers are defined as traits that can be measured as an indicator of a pathogenic process or a pharmacologic response to treatment and can be assessed objectively (1). Enzymatic and immunoassays are the most used methods for quantifying highly abundant biomarkers. However, the rate of introducing novel biomarkers in medicine is reported to be less than two per year (2), and the adoption of these biomarkers in veterinary medicine is often delayed.

Mass spectrometry (MS)-based proteomics has emerged as a powerful technology in biological (3–5) and medical (6, 7) research. It offers the capability to comprehensively characterize the plasma proteome, thereby contributing to the discovery of new biomarkers (8–10). Unlike traditional techniques, mass spectrometry provides high-precision peptide masses and fragmentation spectra derived from sequence-specific digestion of proteins of interest (10). Proteomics is highly specific due to the uniqueness of peptide masses and sequences, in contrast to colorimetric enzyme tests and immunoassays (11).

Among various approaches, liquid chromatography-tandem mass spectrometry (LC-MS/MS) is often a preferred method in medical research due to its high analytical specificity and sensitivity, enabling the detection and quantification of low-abundance proteins, as well as drugs and metabolites (12). Consequently, MS-based proteomic techniques are gaining increasing interest in small animal veterinary medicine. Although research on MS-based proteomic analysis in veterinary medicine is not as extensive as in human clinical research, MS-based studies analyzing biological fluids of dogs with various diseases have been reported (13–19). Given the limited technical and medical data in the veterinary literature, the small number of proteins detected in canine serum in the studies mentioned above, and the importance of proteomic analysis for detecting novel biomarkers or biomarker networks in canine plasma, there is a need to explore new methods for studying blood biomarkers.

Plasma refers to the liquid portion of uncoagulated blood left behind after removing all cell types. Heparin is one of the most used anticoagulants for plasma preparation, acting through anti-thrombin activation. When clot formation in plasma is triggered to form serum, fibrinogen, and other coagulation factors are depleted, while peptides like fibrinopeptide A and B are released (20–22). Over the last decades, the number of proteins identified in human plasma has increased exponentially (23–25). Plasma proteomic analysis offers the advantage of quantifying high-abundance proteins such as fibrinogen and partially other coagulation factors that are no longer present in serum, potentially revealing specific disease patterns. However, due to plasma’s highly high dynamic range, identifying lower abundance proteins by LC-MS can be challenging. Techniques like antibody depletion of abundant plasma proteins such as albumin and fibrinogen, as well as extensive plasma fractionation, have been successfully used to facilitate the detection of low-abundance proteins in human samples (26–28). These techniques have also been combined successfully to identify several thousand proteins (29–31).

Two-dimensional gel electrophoresis (2-DE) has been widely used for proteomic analysis (32, 33). However, this method has limitations in effectively identifying proteins of low abundance, and its dynamic range is limited. Gel-free methodologies have gained attention in recent years since they allow the determination and quantification of a broader range of proteins (34). The need for detecting novel prognostic biomarkers to predict disease outcomes has led proteomic research in canine medicine to focus on infectious diseases, with leishmaniosis being the most prominent example (14, 35, 36), followed by diseases like babesiosis (13, 37–39), ehrlichiosis (40), and parvovirus infection (17). Some studies on patients with leishmaniosis and babesiosis found a significant downregulation of Apolipoprotein A, which may reduce the individual’s capacity to respond to oxidative damage. Beyond the field of infectious diseases, proteomic analysis has provided new insights into veterinary nephrology, revealing that an increase in proteins like retinol-binding protein predicts kidney damage before azotemia develops (41–43), and in veterinary endocrinology, uncovering the role of apolipoprotein I in canine obesity (44, 45). According to the literature, the most analyzed samples in small animal medicine are serum and saliva (46). Studies have also been conducted with other biological fluids and tissues such as cerebrospinal fluid, bile, liver, synovial fluid, myocardium (47–51), and stool samples (52). Currently, there is no systematic catalog of dog plasma proteins available. Although proteomic analysis of canine plasma is less performed than serum analysis, some studies on canine plasma have been reported, with the differential abundant plasma proteins detected in dogs with SIRS and MODS being 68, 12 in obese dogs with and without obesity-related metabolic dysfunction, and finally 87 in dogs diagnosed with canine cognitive dysfunction syndrome, respectively (19, 53, 54).

This study aims to utilize label-free quantification LC-MS proteomic analysis to explore the composition of canine plasma in healthy dogs from different breeds. These findings will serve as a foundation for future studies involving larger disease-specific cohorts, with the goal of detecting new biomarkers or gaining a better understanding of biomarker networks in various diseases.

## Materials and methods

### Animals and sample collection

Clinically healthy client-owned dogs (*N* = 30) with no signs of disease within the last 2 months were presented at the Division for Small Animal Internal Medicine (Veterinary University of Vienna, Vienna, Austria) over 6 months for clinical examination and blood sampling. They were enrolled in a study (Ref: BMBWF 20221-0.210.26) conducted by the Division for Small Animal Internal Medicine and the Interuniversity Messerli Institute of Research (Veterinary University of Vienna, Austria). Before enrollment, written informed consent was obtained from the owners. Dogs of different breeds, body weights, and genders, ranging in age from 1 to 10 years old, were considered for the proteomics study. Inclusion criteria involved a comprehensive evaluation, including a detailed history, physical examination, and blood sampling performed by two authors (PD and CF). Complete blood count (CBC), serum biochemical profile, and electrolyte measurements were conducted. Additionally, the body condition score (Nestle Purina scale: ranging from 1-very thin to 9-significant obesity) of each dog was recorded. Dogs younger than 1 year, weighing less than 5 kg, and those with any pathological signs or recent medication administration within the last 2 months were excluded from the study. Likewise, dogs with significant alterations in any blood parameters were not enrolled. Randomization and group selection were performed using the “tidyverse” R package.

### Sample preparation for proteome analysis

Blood samples were collected using 2 mL Vacuette tubes with lithium heparin 13×75 green cap-white ring PREMIUM (Greiner Bio-One GmbH, Bad Haller Str. 32, 4550, Austria). After centrifugation at 2,000 × g for 5 min, the plasma was separated and stored at −21°C. Five microliters of plasma from each dog of the cohort of 30 individuals were used to create random pools. We created three separate groups that consisted of five pools, each containing pooled plasma from six individuals for a final volume of 30 μL. One of the groups went for 14 more abundant protein depletion procedures using a commercial kit (Top14 Abundant Protein Depletion Mini Spin Columns, Thermo Scientifics, Germany) that was used following the manufacturer’s instructions. The second group consisted of an in-house albumin depletion procedure using Blue-Sepharose CL6B (GE Health Care, Germany). The procedure consisted of mixing 30 μL of plasma pools with 100 μL of pre-equilibrated Blue Sepharose with phosphate buffer (PBS, 150 mM, pH 7.2), incubation for 30 min with soft shaking, separation of the supernatant by centrifugation (4,000 × g for 10 min), and separation of the surplus plasma that was used for the proteomic procedure. The third group consisted in pools of plasma without any depletion treatment that were used directly for proteomics.

Five microliters of plasma per pool was transferred to a tube containing 20 μL of urea denaturing buffer (6 M urea, 2 M thiourea, and 10 mM HEPES, pH 8.0). Disulfide bonds from the plasma proteins were reduced by adding 1 μL of dithiothreitol (10 mM, stock concentration) and incubated for 30 min at room temperature. Afterward, the samples were alkylated by adding 1 μL of iodoacetamide (55 mM, stock concentration) solution and incubated at room temperature for another 30 min in the dark. The samples were diluted with four volumes of ammonium bicarbonate buffer (40 mM) and digested overnight at 37°C by adding 1 μg of trypsin protease (Thermo Scientific, United States). To acidify the samples, 5% acetonitrile and 0.3% trifluoroacetic acid (TFA; final concentration) were added, and subsequently, the samples were desalted using C18 StageTips with Empore^™^ C18 Extraction Disks (55). The peptides eluted from the StageTips were dried using vacuum centrifugation.

### Liquid chromatography-mass spectrometry analysis

Peptides were reconstituted in 40 μL of a solution containing 0.05% TFA and 4% acetonitrile. Then, 2 μL of each sample was applied to an Ultimate 3,000 reversed-phase capillary nano liquid chromatography system connected to a Q Exactive HF mass spectrometer (Thermo Fisher Scientific). The samples were injected and concentrated on a PepMap100 C18 trap column [3 μm, 100 Å, 75 μm inner diameter (i.d.) × 20 mm, nanoViper; Thermo Scientific] that was equilibrated with 0.05% TFA in water. After switching the trap column inline, LC separations were performed on an Acclaim PepMap100 C18 capillary column (2 μm, 100 Å, 75 μm i.d. × 500 mm, nanoViper, Thermo Scientific) at an eluent flow rate of 300 nL/min. Mobile phase A consisted of 0.1% (v/v) formic acid in water, while mobile phase B contained 0.1% (v/v) formic acid and 80% (v/v) acetonitrile in water. The column was pre-equilibrated with 5% mobile phase B, followed by an increase to 44% mobile phase B over 35 min. Mass spectra were acquired in a data-dependent mode, utilizing a single MS survey scan (*m*/*z* 300–1,650) with a resolution of 60,000, and MS/MS scans of the 15 most intense precursor ions with a resolution of 15,000. The dynamic exclusion time was set to 20 s, and the automatic gain control was set to 3 × 10^6^ and 1 × 10^5^ for MS and MS/MS scans, respectively.

### Data processing and label-free quantification

MS and MS/MS raw data were analyzed using the MaxQuant software package (version 2.0.3.0) with the implemented Andromeda peptide search engine (56). The data were searched against the *Canis lupus familiaris* reference proteome (ID: UP000002254; downloaded from Uniprot.org on 17.10.2022; 43,621 sequences) using the default parameters and enabling the options of label-free quantification (LFQ) and match between runs. Data filtering and statistical analysis were conducted using the Perseus 1.6.14 software (57). Only proteins identified and quantified with LFQ intensity values in at least three (out of five) replicates within at least one of the three experimental groups were used for downstream analysis. Missing values were replaced from a normal distribution (imputation) using the default settings (width 0.3, downshift 1.8). Mean log2-fold differences between groups were calculated in Perseus using Student’s t-test. Proteins with a minimum 2-fold intensity change compared to the control (log2-fold change ≥1 or log2-fold change ≤ −1) and a *q*-value ≤0.05 (adjusted *p*-value) were considered significantly abundant.

### Statistical analysis

All statistical comparisons between groups were performed using the student’s t-test implemented by the Perseus computational platform (57). Adjusted *p*-values after FDR (*q*-values) were considered significant for values below 0.05.

## Results and discussion

In our study, we utilized a label-free quantification LC-MS method to analyze canine plasma. The plasma of 30 healthy individuals was used. The median age of the 30 dogs was 5.7 years old (range 1.3–9.9 years old), the median weight was 18.4 kg (range 5–37.5 kg), while 15 dogs were male (50%), and 15 dogs were female (50%). Sixteen (60%) dogs were castrated, and 14 (40%) were intact. All included dogs had an ideal body condition score of 5/9. Of these dogs, 9 were mix breeds, 3 Australian Shepherds, 2 Golden Retrievers, 2 German Shepherds, 2 Pulis and 12 other breeds were represented by one dog each. These samples were randomly selected, according to the inclusion criteria, to avoid preserving heterogenicity and were thereafter grouped into five pools of six individuals using a computer script, as outlined in the material and methods section. We assessed the plasma protein content and evaluated two depletion methods for high-abundance proteins. The first method involved the use of a commercial kit designed for depleting the 14 most abundant human plasma proteins (referred to as “kit”), while the second method employed a low-cost in-house approach using Blue-Sepharose. The purpose of the Blue-Sepharose method was specifically to remove albumin (referred to as “Blue-Sepharose”). To optimize the process, we incorporated a set of clinical samples obtained from healthy individuals with well-established normal profiles of routine blood parameters.

**Figure 1:**
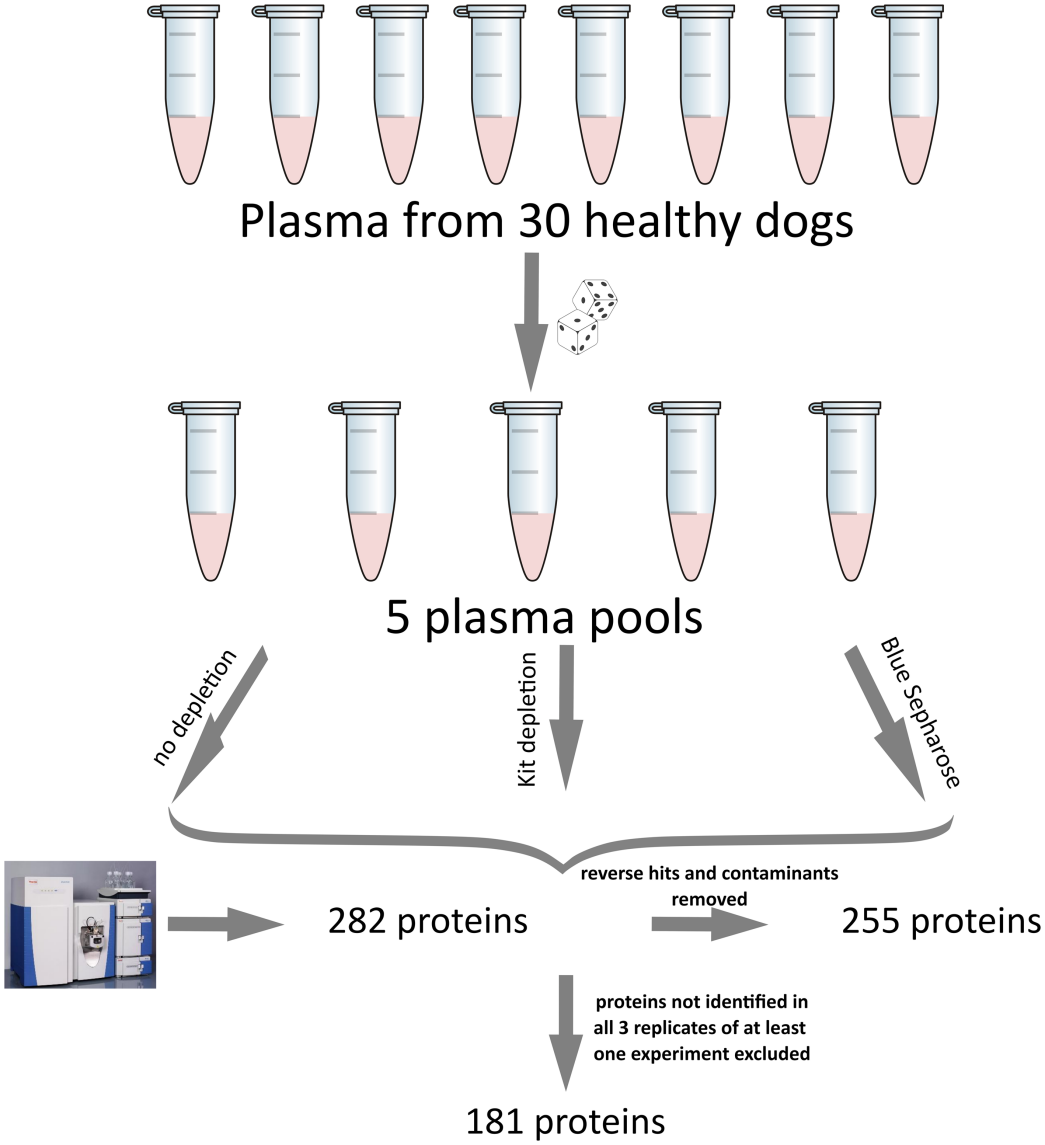
Illustration of the experimental setup and data processing for the canine plasma proteome analysis used in this study. The five pools were generated using a computer script to ensure the randomization of each sample. Initially, 282 proteins were detected. After removing reverse hits and contaminants, analyzed, 255 proteins remained, and after excluding proteins not identified in at least 3 out of 5 replicates of at least one experiment, a total of 181 proteins was analyzed.

All result files from our MS experiments are provided in the supplementary material. Initially, we identified a total of 282 proteins. Subsequently, proteins that were not identified in at least 3 out of 5 replicates from at least one of the three groups (Control, Kit, Blue-Sepharose) removed. This filtering process resulted in the quantification of 181 proteins in the plasma samples. Figure 1 illustrates the above-mentioned data processing steps. For protein description, gene IDs were primarily used. In cases where gene names were unavailable, protein IDs were utilized instead. Among all the samples, some of the most abundant proteins identified were apolipoprotein A and B (APOA, APOB), albumin (ALB), alpha-2-macroglobulin (A2M), fibrinogen beta chain (FGB), fibronectin (FN1), complement C3 (C3), serotransferrin (LOC477072), coagulation Factor V (F5), maltase-glucoamylase (MGAM), and several uncharacterized proteins (LOC611458, LOC481722, A0A8I3P3U9). For a detailed list of all identified proteins and the raw data, please refer to the Supplementary Table S1.

Before excluding proteins that were not detected in at least three replicates from any of the three groups, 163 out of the 181 proteins were present in all three groups. In contrast, 15 proteins were only present in the Control and Kit groups, five proteins to the Control and Blue-Sepharose groups, and 5 proteins to the Kit and Blue-Sepharose groups. Additionally, one protein (S100A12) was exclusively identified in the kit depletion experiment, and two proteins (Ig-like domain-containing proteins, Protein IDs: A0A8I3P3T7 and A0A8I3P941) were identified solely in the Blue-Sepharose depletion experiment, and one protein (AMBP) was exclusively identified in the experiment without depletion (control).

**Figure 2:**
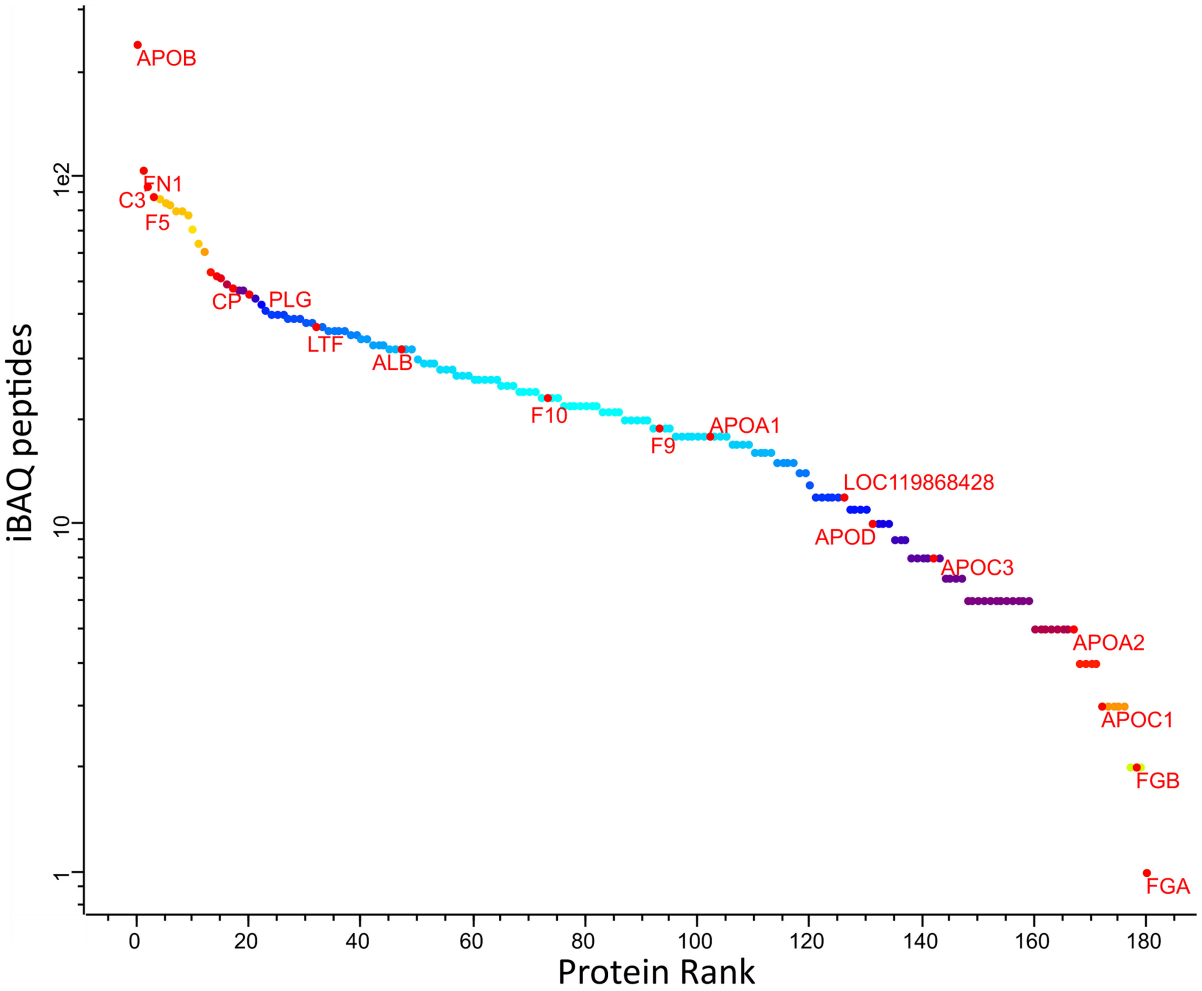
Abundance rank of the identified canine plasma proteins amongst all samples. Selected proteins representing different levels of abundances are labelled: ALB, albumin; APOA1, apolipoprotein A1; HP, haptoglobin; C3, compliment 3; GC, vitamin D-binding protein; APOA4, apolipoprotein A4; APOB, apolipoprotein B; APOC2, apolipoprotein C2; FN1, fibronectin 1; IGFALS, insulin-like growth factor binding protein, Fibrinogen alpha chain; F9, coagulation factor 9; CDH5, cadherin 5.

The ranking of protein abundance can be observed in Figure 2. The principal component analysis demonstrated significant and clear segregation among the three methods (Supplementary Figure S2). The pairwise hierarchical clustering and correlation analysis of all protein samples using the two different depletion methods and the total proteins detected without depletion as control, are depicted in Supplementary Figure S3.

Significant depletion of high-abundance proteins, mainly albumin, was not achieved with either technique (Kit, Blue-Sepharose) compared to the control technique without depletion (Supplementary Figure S1). However, the log2 fold-change of albumin concentration in samples after using the kit and the Blue-Sepharose depletion was small (0.312) but statistically significant (*q-*value = 0.007), with albumin being more abundant after depletion with Blue-Sepharose. One possible explanation for the inadequate depletion of high-abundance canine plasma proteins with the commercial kit is that the kit used in this study is designed for depleting high-abundance proteins in human plasma or serum and has not been validated for depleting proteins from canine plasma. The antibodies used for the kit could not entirely specific and have an affinity for several other proteins (27). Furthermore, the Blue-Sepharose depletion method used in this experiment is not yet standardized for animal samples, and we hypothesize that variations in the protocol, such as the amount of sample or Blue-Sepharose added, might contribute to better depletion results. An old study reported that canine albumin binds with a lower affinity to Blue-Sepharose. We could not notice this before performing this study because the old name of Blue Sepharose was Cibacron Blue (58).

In this study, we used canine plasma instead of serum as we aimed to identify proteins involved in the coagulation cascade. Fibrinogen A (FGA) showed a significant decrease in abundance (log2 fold-change −1.469, *q*-value = 0.006) after depletion with the kit compared to the control group. However, it is still unclear to what extent fibrinogen affects depletion and how it interferes with detecting lower abundant proteins.

**Figure 3:**
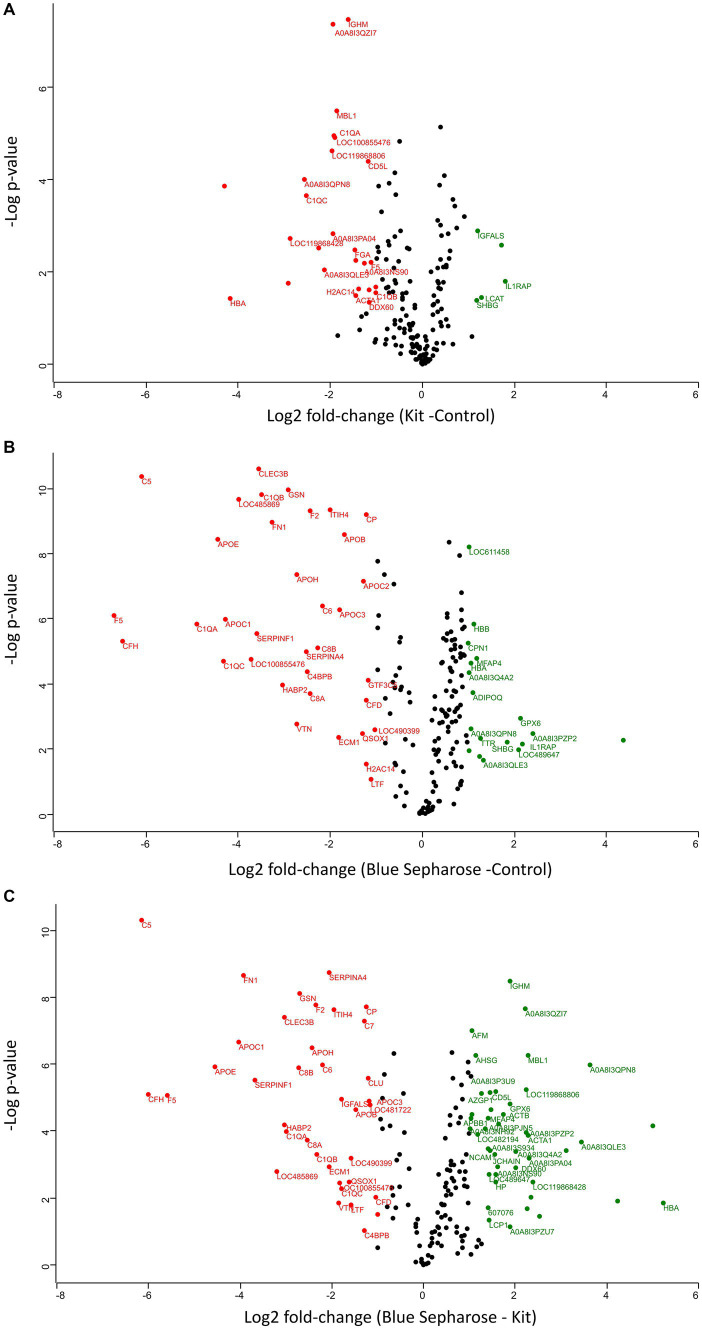
Volcano plots (−log *p*-values versus log2 fold-change) of protein intensity were measured by LC-MS of canine plasma proteins from the three conditions of plasma processing in this study **(A–C)**. Proteins with a minimum 2-fold intensity change compared to the control (log2 fold-change ≥1 or log2 fold-change ≤ −1) and a *q*-value ≤0.05 were considered significantly abundant. Black dots represent non-significant differentially abundant proteins, green dots show the up-represented fraction and, and red ones represent the down-represented fraction.

The two different depletion methods exhibit significant differences in the fold change of numerous proteins compared to the three techniques (Figures 3A–C). Thirty-two proteins were differentially abundant among the control and kit depletion methods, with 27 being more abundant with the control method and 5 with the kit depletion method (Supplementary Table S2). Among the most important proteins that showed a significant increase after kit depletion compared to the control method are interleukin-1 receptor accessory protein (IL1RAP), solute carrier family 12 member 4 (LCAT), insulin-like growth factor binding protein acid labile subunit (IGFALS), sex hormone-binding globulin (SHBG), and V-type proton ATPase subunit G (A0A8I3PF02). On the other hand, hemoglobin subunit alpha (HBA), ferritin (LOC119868428), complement C1q C (CIQC), fibrinogen alpha chain (FGA), and Ig-like domain-containing protein (A0A8I3PB96) were significantly more abundant in the control experiment without depletion (Supplementary Figure S4A). Fifty-three proteins were differentially abundant among the control and Blue-Sepharose depletion methods, with 35 being more abundant with the control method and 18 with the Blue-Sepharose depletion method (Supplementary Table S3). Interestingly, IL1RAP and SHBG exhibited a significantly increased relative intensity after depletion with the Blue-Sepharose method compared to the control group (Supplementary Figure S4B). Finally, we directly compared the proteins detected with the two different depletion techniques (Supplementary Figure S4C). Eighty-two proteins were differentially abundant among the Blue-Sepharose and kit depletion methods, with 45 being more abundant with the Blue-Sepharose method and 37 with the kit depletion method (Supplementary Table S4). The most noteworthy proteins that were significantly more abundant after kit depletion include complement C5 (C5), coagulation Factor V (F5), apolipoprotein E (APOE), fibronectin (FN1), and serpin family F member (SERPINF1), while HBA, Ig-like domain-containing protein (A0A8I3QPN8), ferritin (LOC119868428), C-type lectin domain-containing protein (MBL1), and immunoglobulin heavy constant mu (IGHM) were found in significantly higher concentrations using the Blue-Sepharose method.

In our experimental conditions, we did not find a clear benefit of using depletion methods because one of the goals of using these methods was to increase the number of detected proteins, which was not achieved in our study. There is a possibility that the kit for protein depletion is not well-optimized for dog plasma. This seems to be the case for Blue-Sepharose as well, as albumin did not significantly decrease, and other unexpected proteins were decreased, likely due to nonspecific binding. For this reason, with the present instrumental setup described here, we consider that it is not necessary, in principle, to use a depletion method for canine plasma analysis, or alternatively, a new specific depletion method should be developed and tested. In the case of Blue-Sepharose, the diminishing abundance of specific proteins, but not albumin, may indicate nonspecific and unpredictable binding of these protein sets. In contrast, albumin unexpectedly did not decrease in concentration.

A recent report indicates the quantification of around 400 plasma proteins using different fractionation techniques (59). We also have recently successfully used the methodology described in this work to uncover the plasma proteome signature of canine acute hemorrhagic diarrhea syndrome (AHDS). In that study, we detected a similar number of proteins as reported here. For instance, we found that serpina3, lipopolysaccharide-binding protein, glyceraldehyde-3-phosphate dehydrogenase, and serum amyloid A were more abundant in plasma from AHDS-affected dogs. In contrast, other proteins, such as paraoxonase, selenoprotein, amine oxidases, and apolipoprotein C-IV, were significantly less abundant. Many identified and quantified proteins were known to be associated with inflammation, validating the ability of this method to detect disease-related biomarker candidates in veterinary medicine (60).

## Conclusion

LC-MS analysis using label-free quantification can reliably detect and quantify multiple proteins in canine plasma, making it a valuable tool for unraveling the pathogenesis of various diseases in veterinary medicine. As a tool, it provides influential information for accurate disease diagnosis and prognosis estimation in future analyses. Our results indicate that protein depletion with the two methods described herein is not adequately achieved. Although the abundance of various proteins can differ significantly, these methods, as performed here, do not contribute to the determination of lower abundance proteins in canine plasma. Prospective controlled studies in animal disease models are expected to shed light on the utility of gel-free label-free LC-mass spectrometry proteomic analysis in veterinary medicine.

## Data availability statement

The data presented in the study are deposited in the Phaidra repository, accession number 2720; Proof identifier-https://phaidra.vetmeduni.ac.at/o:2720 and are furhtermore provided as Supplementary material.

## Ethics statement

The animal study was approved by Ethics Commission of the University of Veterinary Medicine. The study was conducted in accordance with the local legislation and institutional requirements.

## Author contributions

PD: Conceptualization, Formal analysis, Investigation, Methodology, Software, Writing – original draft, Writing – review & editing. BK: Data curation, Formal analysis, Methodology, Resources, Software, Validation, Writing – original draft, Writing – review & editing. CF: Data curation, Investigation, Writing – original draft, Writing – review & editing. AR-R: Conceptualization, Data curation, Formal analysis, Investigation, Methodology, Project administration, Resources, Software, Supervision, Validation, Visualization, Writing – original draft, Writing – review & editing. IB: Conceptualization, Funding acquisition, Investigation, Methodology, Resources, Supervision, Validation, Visualization, Writing – original draft, Writing – review & editing.
