# Supplementary material for: Characterization of the plasma proteome from healthy adult dogs
Source: Front Vet Sci. 2024 Apr 4;11:1356318. doi: 10.3389/fvets.2024.1356318 (PMC11024428; doi:10.3389/fvets.2024.1356318)
Supplement: Supplementary file 1 [file Data_Sheet_1.pdf]

Supplementary Material

"Characterization of the plasma proteomefrom healthy adult dogs"

Pavlos G. Doulidis[1], Benno Kuropka[2], Carolina Frizzo Ramos[3], Alexandro Rodríguez-Rojas[1], Iwan A. Burgener[1*].

[1] Division for Small Animal Internal Medicine, Department for Small Animals and Horses, University of Veterinary Medicine Vienna, Vienna, Austria.

[2] Protein Biochemistry, Institute of Chemistry and Biochemistry, Freie Universität Berlin, Berlin, Germany.

[3] The Interuniversity Messerli Research Institute, Medical University Vienna, and University of Veterinary Medicine Vienna, Vienna, Austria.

*Correspondence: Iwan.Burgener@vetmeduni.ac.at

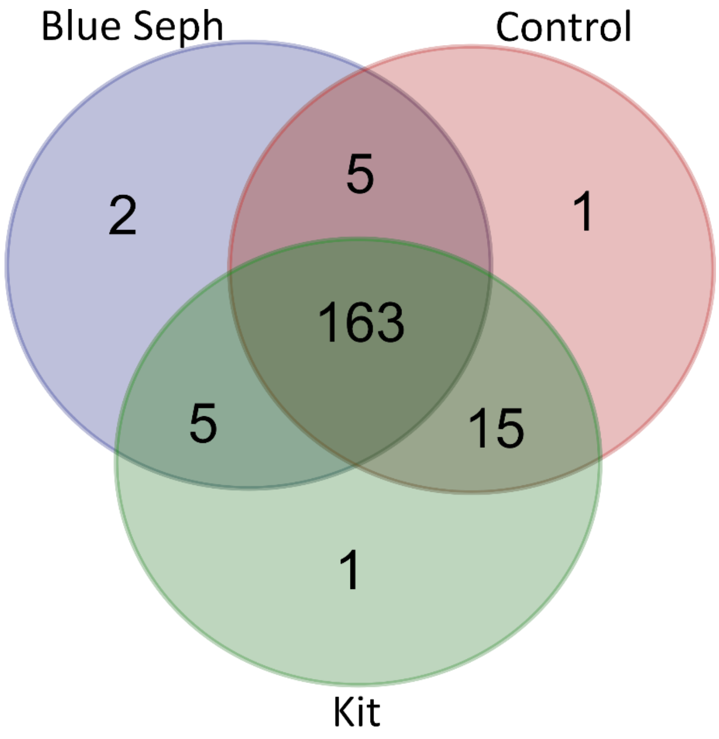

Supplementary Figure S1. Venn diagram of differentially abundant proteins of plasma from Control, Kit, and Blue-Sepharose groups.

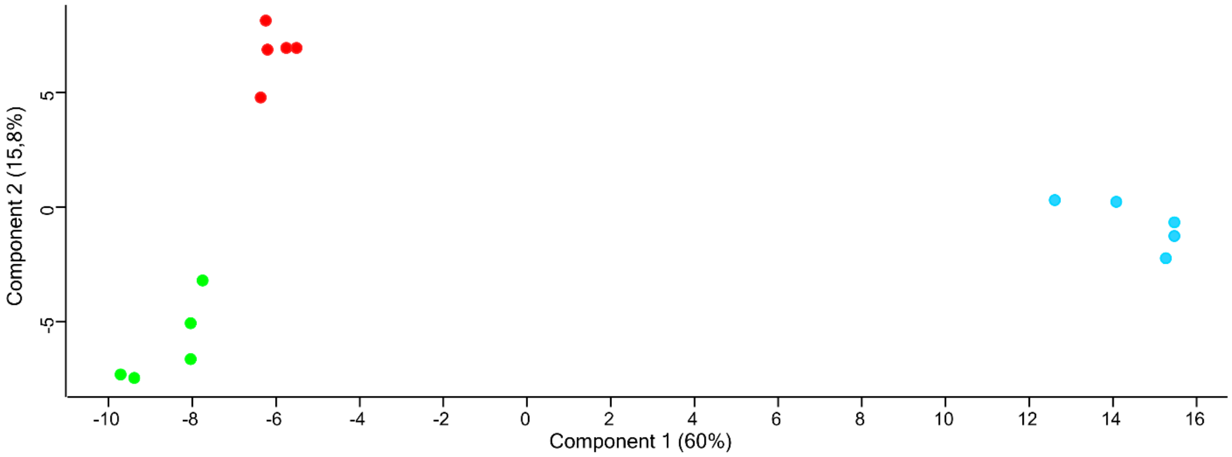

Supplementary Figure S2. Principal Component Analysis of the three different methods used. A clear segregation can be seen between the three groups. (Control in red, Kit in green, and Blue-Sepharose 23 in light blue).

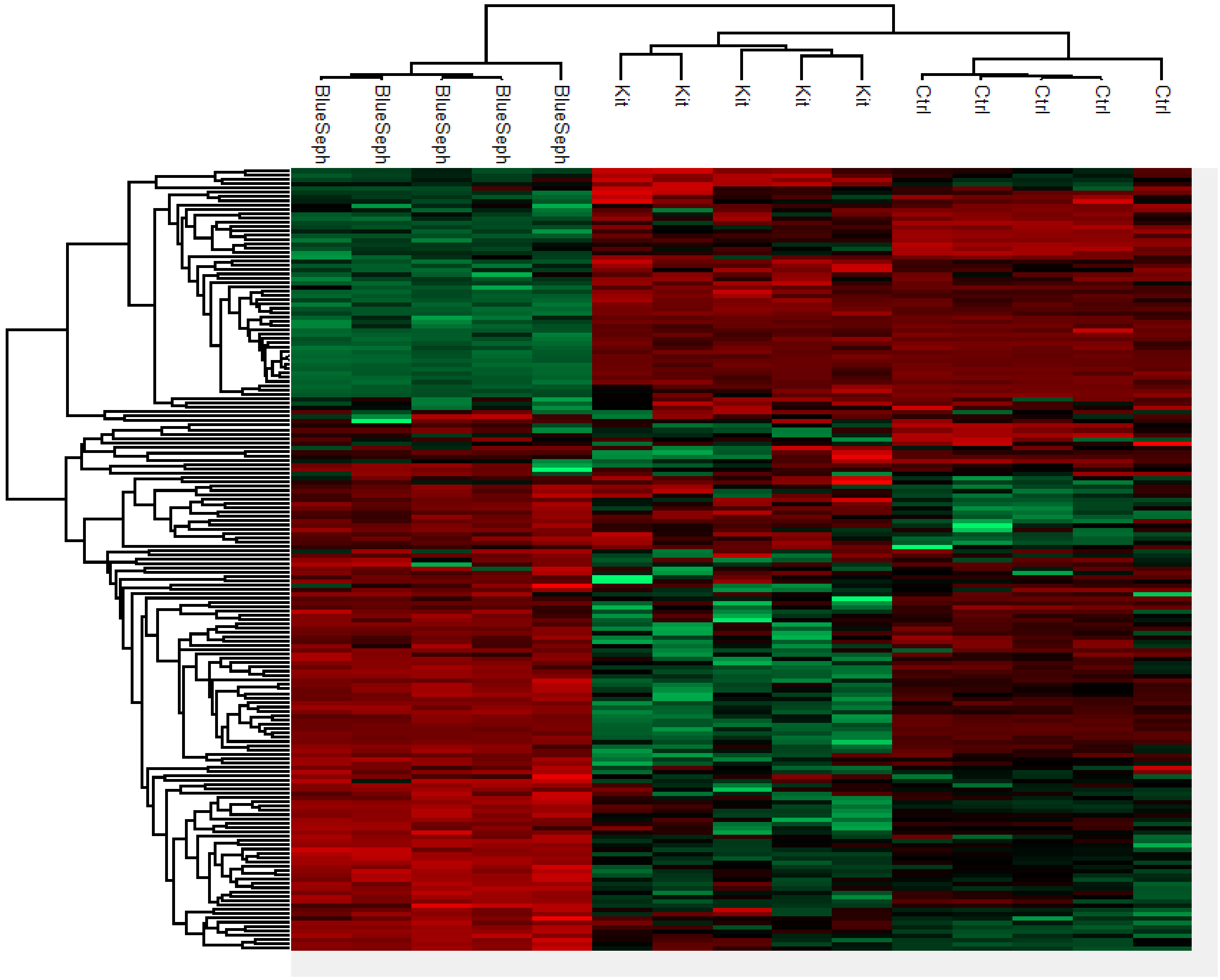

Supplementary Figure S3. Heatmap of the detected proteins, presenting the result of a two-way hierarchical clustering of the proteins found in the three groups. The diagram was constructed using the complete-linkage method together with the Euclidean distance. Each row represents a differentially abundant proteins and the columns are the different samples tested (5 pools in each group). The intensity scale illustrates the relative level of differentially protein concentrations with green portraying up-represented and red down-represented.

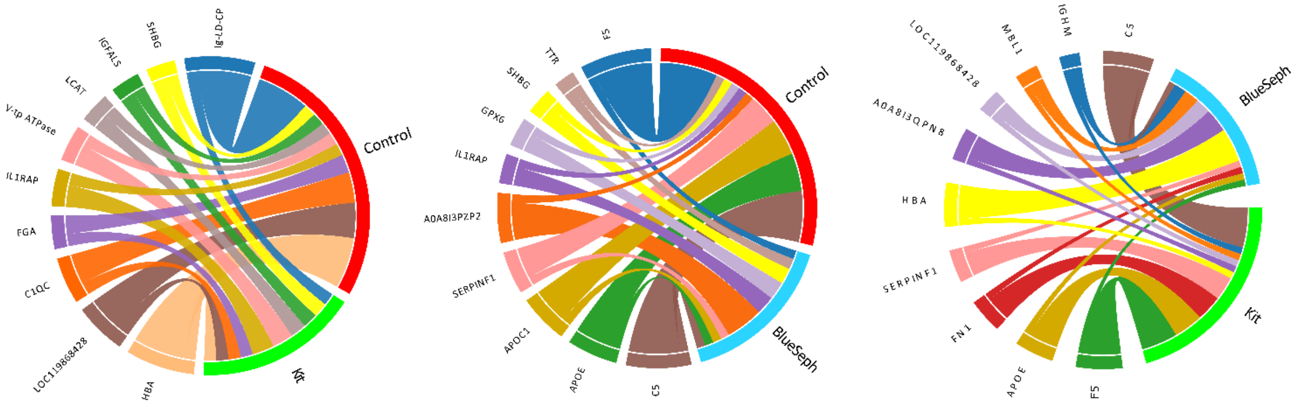

A B C

Supplementary Figure S4 A, B, C. Chord diagrams showing the associations among differentially abundant proteins of Control, Kit, and Blue-Sepharose (Blue Seph) groups. Only the most important abundant proteins detected are shown. For better comprehension, each circle includes two comparisons each time (Control in red, Kit in green, and Blue-Sepharose in light blue). The chord diagrams show the key proteins identified by their comparative abundance. The outer ribbon identifies the respective groups-experiment and encompasses the perturbed protein quantification with each method. Chords connect proteins related to more than one method in the inner circle. Only significant hits are represented in these chord charts (at least q<0.05).

Legends to the Supplementary Tables

Supplementary Table S1. Output table of the proteomic experiment reporting plasma protein detection and quantification from dog plasma. The data includes treating three conditions: total plasma, depletion fraction (Thermo Scientific depletion kit), and in-house albumin depletion using Blue-Sepharose. The same procedure was applied to five dog plasma pools (six individuals each). Statistical analysis used student t-test and false discovery rate (FDR) to correct the p-values (data analysis using MaxQuant and Perseus software for label-free quantification of proteins with LC-MS).

Supplementary Table S2. Table showing the differentially abundant proteins among Control and Kit methods including protein ID, Gene name, fold-change and statistical significance.

Supplementary Table S3. Table showing the differentially abundant proteins among Control and Blue Sepharose methods including protein ID, Gene name, fold-change and statistical significance.

Supplementary Table S4. Table showing the differentially abundant proteins among Blue Sepharose and Kit methods including protein ID, Gene name, fold-change and statistical significance.
